# Supplementary material for: Multiplexed CRISPRi reveals a transcriptional switch between KLF activators and repressors in the maturing neocortex
Source: eLife. 2026 Oct 20;14:RP106510. doi: 10.7554/eLife.106510 (PMC13682713; doi:10.7554/eLife.106510)
Supplement: Peer review history

# Editor report: eLife Assessment

**DOI:** 10.7554/eLife.106510.3.sa0

**Authors:** Joshua Corbin

**Affiliations:** Children's National Hospital United States

**Keywords:** Compelling, Important

This is an **important** study utilizing innovative CRISPR based approaches demonstrating the role of the KLF family of transcription factors in the post-natal maturation of cortical projection neurons. The strength of evidence overall is **compelling**, and the study is well executed. The screen data presented provides a number of interesting candidates for future analyses into the mechanism of action of KLF family members in neuronal maturation.

# Referee report: Reviewer #1 (Public review)

**DOI:** 10.7554/eLife.106510.3.sa1

[Editors' note: this version has been assessed by the Reviewing Editor without further input from the original reviewers. The original reviews were generally positive. In their revision, the authors partially address reviewers concerns, but mention that a number of requested experiments are for future studies.]

Summary:

This is an interesting manuscript by Kirk and colleagues describing a highly valuable knock-down system that leverages CRISPRi in order to further elucidate the role of the Kruppel-Like Factor (KLF) transcription factor family in regulating the maturation of postnatal cortical projection neurons. The authors firstly use RNA-Seq and ATAC-Seq data in order to identify the KLF TF family as a potential regulator of cortical neuron maturation in the postnatal brain and subsequently knock down four KLF family members; KLF9, KL13, KLF6 and KLF7, in order to ascertain the functions of specific KLF genes in the developing cortex. The described CRISPRi knock down strategy is highly robust and penetrant as evidenced by a KD efficiency > 95% (assessed by both qPCR and single molecule FISH) and demonstrates that KLF6 and KLF7 play an activating role in driving the expression of target genes relating to axonal growth whereas KLF9 and 13 play a repressive role that inhibits the expression of overlapping gene targets. Together, the authors propose a model where the KLF TF family acts as a regulatory "switch" from activation to repression in the postnatal cortex as a mechanism to control a shift in projection neuron function from axonal growth to circuit refinement. The findings and conclusions of the manuscript offer a valuable contribution to the field of postnatal cortical development and further our understanding of the regulatory mechanisms that govern neuron maturation.

# Referee report: Reviewer #2 (Public review)

**DOI:** 10.7554/eLife.106510.3.sa2

Summary:

Kirk et al. use RNA-Seq and CRISPRi to provide evidence that KLF family transcription factors regulate postnatal neuronal maturation of pyramidal neurons. The genetic programs regulating postnatal neuronal maturation are not well understood. The authors first analyzed chromatin accessibility and gene expression data from layer 4 and 6 pyramidal neurons and found that KLF TFs are predicted regulators of postnatal neuronal maturation. They then use CRISPRi knockdown and find that KLF activators first activate genes and then this is followed by KLF repressors repressing genes. Interestingly, some genes, such as those with cytoskeletal functions, are shared targets of KLF activators and repressors.

Strengths:

The study is well-executed and the paper is well-written. A major strength of this study is the application of state-of-the-art transgenic approaches. The CRISPRi approach used to knock down multiple KLFs is compelling. The genomic data generated appears to be high quality and is carefully analyzed. The presented findings provide important insights into the genetic programs that regulate postnatal maturation in cortical pyramidal neurons. The discovery that KLF family activators/repressors regulate gene expression changes during this critical step of neuronal development fills an important gap in the field.

Weaknesses:

While beyond the scope of the current study, future studies should investigate the contributions of KLFs on postnatal morphological and physiological changes.

# Referee report: Reviewer #3 (Public review)

**DOI:** 10.7554/eLife.106510.3.sa3

Summary:

In their manuscript "Multiplexed CRISPRi Reveals a Transcriptional Switch Between KLF Activators and Repressors in the Maturing Neocortex", Kirk and colleagues seek to dissect the developmentally regulated pan-neuronal gene programs that control the postnatal maturation of cortical neurons. For this, the authors analyzed newly generated and existing RNA-seq and ATAC-seq of Layer 4 and Layer 6 cortical pyramidal neurons at postnatal day 2 (P2) and day 30 (P30) and identified thousands of shared developmentally regulated genes and genomic (promoter) regions, including genes involved in axon growth (tend to be downregulated) and synaptic function (tend to be upregulated). Motif enrichment analysis of promoters of differentially regulated genes revealed a strong presence of KLF/Sp family binding motifs, pointing to Krüppel-Like Factors (KLFs) as key transcriptional regulators of cortical maturation. Expression profiling showed a developmental switch from activating KLFs (Klf6, Klf7) expressed neonatally to repressive KLFs (Klf9, Klf13) upregulated during maturation. Using an elegant in vivo multiplexed CRISPR interference (CRISPRi) system, the authors achieved efficient, cell-type-specific knockdown of these TFs and showed that Klf9 and Klf13 repress a set of genes that includes cytoskeletal regulators such as Tubb2b, Dpysl3, and Rac3. Conversely, Klf6 and Klf7 promoted the expression of these same genes in the early postnatal period, and their knockdown led to reduced expression of these genes, particularly at P10 when their activating influence is strongest. Since promoters of shared KLF targets were enriched for KLF/Sp motifs but showed little change in chromatin accessibility, the authors propose a model in which distinct KLF family members function either as transcriptional repressors and activators that compete at constitutively accessible promoters and thereby act as a developmental transcriptional switch that coordinates the downregulation of axon growth programs and upregulation of synaptic maturation genes during cortical development.

Strengths:

The study addresses an interesting question and advances our understanding of the transcriptional regulation underlying postnatal cortical development. A major strength of the study lies in the innovative use of in vivo multiplexed CRISPR interference (CRISPRi), which allows for cell-type-specific, combinatorial knockdown of redundant TFs - this an elegant solution to a long-standing challenge in transcription factor research and should be useful also for other neuroscience studies that require local and cell-type-specific gene loss-of-function. Also, the integration of RNA-seq and ATAC-seq across developmental time points provides a robust foundation for identifying direct targets of the KLF family, and the findings are reinforced by cross-species conservation and the identification of targets with clear neurodevelopmental relevance.

Weaknesses:

The major weakness of the study lies in its relatively narrow scope: the study focuses primarily on transcriptional mechanisms and largely lacks functional validation of the neuronal phenotypes that are predicted by the gene expression data (e.g. axonal morphology). Despite these limitations, the paper offers an interesting model for a transcriptional switch during neuronal maturation in the cortex and establishes a powerful methodological framework for dissecting redundant gene networks in vivo.

# Author comment: Author response

**DOI:** 10.7554/eLife.106510.3.sa4

**Authors:** Ryan Kirk, Liwei Sun, Ruixuan Xiao, Erin A Clark, Sacha B Nelson

**Affiliations:** Brandeis University Waltham United States; Brandeis University Waltham United States; Brandeis University Waltham United States; Brandeis University Waltham United States; Brandeis University Waltham United States

The following is the authors’ response to the original reviews.

**Public Reviews:**

**Reviewer #1 (Public review):**

Summary:

This is an interesting manuscript by Kirk and colleagues describing a highly valuable knock-down system that leverages CRISPRi in order to further elucidate the role of the Kruppel-Like Factor (KLF) transcription factor family in regulating the maturation of postnatal cortical projection neurons. The authors firstly use RNA-Seq and ATAC-Seq data in order to identify the KLF TF family as a potential regulator of cortical neuron maturation in the postnatal brain and subsequently knock down four KLF family members; KLF9, KL13, KLF6 and KLF7, in order to ascertain the functions of specific KLF genes in the developing cortex. The described CRISPRi knock down strategy is highly robust and penetrant as evidenced by a KD efficiency > 95% (assessed by both qPCR and single molecule FISH) and demonstrates that KLF6 and KLF7 play an activating role in driving the expression of target genes relating to axonal growth whereas KLF9 and 13 play a repressive role that inhibits the expression of overlapping gene targets. Together, the authors propose a model where the KLF TF family acts as a regulatory "switch" from activation to repression in the postnatal cortex as a mechanism to control a shift in projection neuron function from axonal growth to circuit refinement. The findings and conclusions of the manuscript offer a valuable contribution to the field of postnatal cortical development and further our understanding of the regulatory mechanisms that govern neuron maturation.

The conclusions of this manuscript are generally supported by the data, but some aspects of the data collection and analysis require some further clarification. Specifically:

(1) The authors comprehensively assess the molecular effects of KLF TF knock-down, however, the authors do not deeply address the cellular effects of these knock-downs. The authors conclude that knockdown of KLF6/7 and KLF9/13 cause downregulation and upregulation, respectively, of a common set of genes involved in cytoskeletal or axon regulation such as Tubb2 and Dpysl3. How is the morphology of the cells affected by these knockdowns? For example, does KLF9/13 knockdown cause neurite/axonal outgrowth? The authors should perform some basic experiments to assess changes in cell morphology following KLF TF KD. This is the one key point that needs addressing, in my opinion.

We appreciate this comment and agree that the cellular effects of KLF activator and/or repressor KD are not addressed by the experiments in this manuscript. However, the effects of KLF9, KLF13, and KLF9/13 knockdown on neurite outgrowth have been previously examined in vitro (Avci et al., 2012; Avila-Mendoza et al., 2020) and in vivo (Apara et al., 2017). The collective findings of these papers (enhanced neurite outgrowth and axon regeneration following KLF repressor KD) align with the predicted outcome of upregulating the set of cytoskeletal remodeling genes identified as putative KLF family targets in this manuscript. Furthermore, the in vitro results demonstrate that the partial redundancy between KLF9 and KLF13 we identified at the transcriptional level is relevant for their roles in repressing neurite outgrowth. Our results suggest that these previous findings are likely to hold true in cortical neurons in vivo while offering a molecular explanation for these effects at the level of gene expression. Other groups have examined the effect of KLF6 or KLF7 overexpression on corticospinal axon regeneration in vivo and found that these transcription factors can individually promote axon regrowth after injury (Blackmore et al., 2012; Wang et al., 2018). Similarly, this earlier work did not identify transcriptional mechanisms underlying the observed effect so our results also offer a plausible set of targets that could mediate the link between KLF activator overexpression and axon regeneration.

(2) The authors identify 374 DEGs in P10 Klf6/7 KD neurons and 115 DEGs at P20 (figure 6B). Have the authors looked to see what proportion of these DEGs are upregulated in the KLF9/13 KDs in order to get a more global understanding of the degree of overlap in the genes regulated by the KLF family members? [MOU2] Along similar lines, the authors later indicate that there are 144 shared targets between the KLF activator and repressor pairs (Figure 7C). What percentage does this represent of the total number of DEGs between the KLF pairs. This could further illustrate the degree to which the KLF pairs regulate the same set of genes. If it is already indicated in the manuscript, it should be made a bit more clear to the reader.

We thank the reviewer for the suggestion to include more comprehensive quantitative measures of the degree of overlap between targets of KLF activator and repressor pairs. We have included the exact number and percentage of P10 and P20 KLF6/7 targets that are differentially expressed (adj. p-value <= 0.05) in KLF9/13 KD neurons and vice versa in the text in the section associated with Figure 7, which is devoted to describing this class of overlapping targets. Furthermore, figures 7D and S7.2B both show how the full set of KLF6/7 targets are affected by Klf9/13 KD and vice versa. Collectively, this demonstrates that both KLF activator and repressor targets trend towards opposite regulation by the opposing pair.

(3) Figures 5B and 6D2 are very interesting as they relate the changes in gene expression over time in neurons from P2 to P30 to the functions of KLF9/13 and KLF6/7, respectively. I would be curious to see how these two forms of analyses overlap with one another. For example, in Figure 6D2, where would the KLF9/13 upregulated genes fall on the plot shown in Figure 6D2? And would those overlapping genes fit a similar correlation?

We agree with the reviewer that this is a powerful way to visually demonstrate the overlap between KLF6/7 and KLF9/13 targets in a more unbiased way and within a developmental context. The suggested analysis has been included in the supplement to Figure 7 (Fig S7.3).

(4) Figure 7E shows expression levels of shared KLF TF targets in control or KD conditions. Interestingly, the expression of Tubb2b, shows higher expression in ScrGFP P10 when compared to KLF9/13 P20, suggesting that derepression of KLF9/13 does not fully restore the expression level of Tubb2b seen at P10. This may suggest that other repressive regulators may be involved in the downregulation of Tubb2b from P10 to P20[MOU4] . Can the authors further comment on this, perhaps in the discussion, and speculate if there are other regulatory factors at play that may be controlling some of the shared targets by KLF6/7 and KLF9/13?

We thank the reviewer for this keen observation. We have included a comment on this within the text associated with Figure 7. While other regulatory factors are plausible, this is easily explained by low expression of Klf6 and Klf7 in the P20 cortex, which cannot drive transcription of Tubb2b and other targets to the same level as what is observed at P10 when the KLF activators are still relatively abundant, even after repression by Klf9/13 is removed. Within this framework, overexpression of KLF activators on a KLF repressor background would be the only way to restore expression of Tubb2b to its P10 expression levels. This is wholly compatible with our model of ‘push-pull’ regulation of KLF targets outlined in Figure 9. The finding could also be affected by the addition of repressive histone marks by the KLF9/13-associated SID complex in early neonatal development that may persist following the knockdown of these repressors, preventing complete restoration of neonatal expression patterns.

**Reviewer #2 (Public review):**

Summary:

Kirk et al. use RNA-Seq and CRISPRi to provide evidence that KLF family transcription factors regulate postnatal neuronal maturation of pyramidal neurons. The genetic programs regulating postnatal neuronal maturation are not well understood. The authors first analyzed chromatin accessibility and gene expression data from layer 4 and 6 pyramidal neurons and found that KLF TFs are predicted regulators of postnatal neuronal maturation. They then use CRISPRi knockdown and find that KLF activators first activate genes and then this is followed by KLF repressors repressing genes. Interestingly, some genes, such as those with cytoskeletal functions, are shared targets of KLF activators and repressors.

Strengths:

The study is well-executed and the paper is well-written. A major strength of this study is the application of state-of-the-art transgenic approaches. The CRISPRi approach used to knock down multiple KLFs is compelling. The genomic data generated appears to be high quality and is carefully analyzed. The presented findings provide important insights into the genetic programs that regulate postnatal maturation in cortical pyramidal neurons. The discovery that KLF family activators/repressors regulate gene expression changes during this critical step of neuronal development fills an important gap in the field.

Weaknesses:

A limitation of the current study is that the functional importance of KLF for postnatal neuronal maturation is unclear. Although the authors find that KLFs regulate some of the gene expression changes during postnatal neuronal maturation, it is still unclear whether such gene expression changes mediate the postnatal changes in morphology and physiology. While beyond the scope of the current study, future studies should investigate the contributions of KLFs on postnatal morphological and physiological changes.

We thank the reviewer for their helpful comments on this manuscript. We agree that the effects of KLF knockdown identified in this study are primarily descriptive, but – as noted - a detailed analysis of the morphological and physiological consequences of KLF knockdown are beyond the scope of this paper. However, we believe that a more mechanistic model of KLF function during neuronal maturation can be obtained by considering our findings on the bidirectional regulation of core cytoskeletal genes by KLF activators and repressors alongside published in vivo and in vitro data on the opposing roles of KLF family members on axon outgrowth/regrowth (Apara et al., 2017; Avila-Mendoza et al., 2020; Blackmore et al., 2012; Moore et al., 2009; Wang et al., 2018). Thus, we offer a set of transcriptional targets that likely mediate these opposing effects and a developmental context within which they might operate.

**Reviewer #3 (Public review):**

Summary:

In their manuscript "Multiplexed CRISPRi Reveals a Transcriptional Switch Between KLF Activators and Repressors in the Maturing Neocortex", Kirk and colleagues seek to dissect the developmentally regulated pan-neuronal gene programs that control the postnatal maturation of cortical neurons. For this, the authors analyzed newly generated and existing RNA-seq and ATAC-seq of Layer 4 and Layer 6 cortical pyramidal neurons at postnatal day 2 (P2) and day 30 (P30), and identified thousands of shared developmentally regulated genes and genomic (promoter) regions, including genes involved in axon growth (tend to be downregulated) and synaptic function (tend to be upregulated). Motif enrichment analysis of promoters of differentially regulated genes revealed a strong presence of KLF/Sp family binding motifs, pointing to Krüppel-Like Factors (KLFs) as key transcriptional regulators of cortical maturation. Expression profiling showed a developmental switch from activating KLFs (Klf6, Klf7) expressed neonatally to repressive KLFs (Klf9, Klf13) upregulated during maturation. Using an elegant in vivo multiplexed CRISPR interference (CRISPRi) system, the authors achieved efficient, cell-type-specific knockdown of these TFs and showed that Klf9 and Klf13 repress a set of genes that includes cytoskeletal regulators such as Tubb2b, Dpysl3, and Rac3. Conversely, Klf6 and Klf7 promoted the expression of these same genes in the early postnatal period, and their knockdown led to reduced expression of these genes, particularly at P10 when their activating influence is strongest. Since promoters of shared KLF targets were enriched for KLF/Sp motifs but showed little change in chromatin accessibility, the authors propose a model in which distinct KLF family members function either as transcriptional repressors and activators that compete at constitutively accessible promoters and thereby act as a developmental transcriptional switch that coordinates the downregulation of axon growth programs and upregulation of synaptic maturation genes during cortical development.

Strengths:

The study addresses an interesting question and advances our understanding of the transcriptional regulation underlying postnatal cortical development. A major strength of the study lies in the innovative use of in vivo multiplexed CRISPR interference (CRISPRi), which allows for cell-type-specific, combinatorial knockdown of redundant TFs - this an elegant solution to a long-standing challenge in transcription factor research, and should be useful also for other neuroscience studies that require local and cell-type-specific gene loss-of-function. Also, the integration of RNA-seq and ATAC-seq across developmental time points provides a robust foundation for identifying direct targets of the KLF family, and the findings are reinforced by cross-species conservation and the identification of targets with clear neurodevelopmental relevance.

Weaknesses:

The major weakness of the study lies in its relatively narrow scope: the study focuses primarily on transcriptional mechanisms and largely lacks functional validation of the neuronal phenotypes that are predicted by the gene expression data (e.g. axonal morphology). For example, the authors analyzed the effects of KLF9/13 KD on the neurons' excitability and excitatory inputs, but did not assess the effects on inhibitory inputs and E/I-ratio or morphological parameters such as axonal length and axonal target fields - the manuscript would be strengthened considerably by such analyses (axonal projections could be analyzed e.g. via local injections of the gRNA AAVs and subsequent immunolabeling of brain sections). Similarly, the chromatin-based mechanisms underlying KLF activity remain relatively speculative, and the transcriptional mechanisms upstream of the KLFs remain unexplored (this could be addressed by analyzing existing datasets; see "Additional Point 1" below). Finally, the manuscript is too long (e.g., nearly five pages in the Discussion section are devoted to discussing various misregulated genes) and would benefit from presenting the Results and Discussion sections more concisely. However, despite these limitations, the paper offers an interesting model for a transcriptional switch during neuronal maturation in the cortex and establishes a powerful methodological framework for dissecting redundant gene networks in vivo.

Shorten discussion (possibly results also).

We appreciate the reviewer’s feedback and agree that linking transcriptional perturbations to cellular phenotypes of KLF activator and/or repressor KD would significantly strengthen this manuscript. However, we believe this is beyond the scope of this current manuscript and the effects of individual KLF activators and repressors on axon outgrowth/regrowth are known from prior in vitro and in vivo studies (Apara et al., 2017; Avila-Mendoza et al., 2020; Blackmore et al., 2012; Moore et al., 2009; Wang et al., 2018). Since there was no detectable effect of KLF repressor KD on excitatory synaptic transmission (Fig. S4.2), we did not believe it was likely that our excitatory neuron-specific knockdown would affect inhibitory synapses under our model where KLF targets have primarily axonal functions. In the absence of ATAC-seq from Klf9/13 KD neurons, the relationship between chromatin accessibility and KLF binding is limited to descriptions of chromatin accessibility around KLF targets in neonatal and mature neurons. However, we do offer two testable hypotheses in our Discussion of early developmental transitions driving the KLF switch (Thyroid Hormone and structured activity input). Finally, we recognize that the manuscript is too long and have edited or removed parts of the Discussion.

**Recommendations for the authors:**

Summary of Recommendations from Reviewing Editor: While the screen data is important and novel, there is consensus among the reviewers that the current study would greatly benefit by additional analyses as to the consequences of KLF knockdown on neuronal morphology and neurite outgrowth. It is recommended that this major open question be experimentally addressed. It is also recommended that additional comments/points below are addressed with changes to the figures/text as appropriate.

**Reviewer #1 (Recommendations for the authors):**

Minor points:

(1) In Figure 2C2, it can be assumed that green represents KLF7 and red represents KLF9 but a legend indicating this would be helpful here. The Y axis is labeled as "Binned D-V Axis". Can the authors further clarify what this means? Is it referring to the different layers of the cortex? It looks like the expression pattern of KLF7 and 19 is not consistent throughout the entire dorsal-ventral axis at P7 and there is a region where KLF19 expression is higher than KLF7. Could this suggest that KLF family members may act on different types of neurons in distinct layers at different timepoints? Could the authors expand on this at all?

We thank the reviewer for pointing this out and the appropriate legend has been added. A detailed description of our method for binning counts in the Dorsal-Ventral axis (y-axis) to normalize for differences in cortical thickness across ages is included in the methods section (see RNAScope Image Acquisition and Analysis), but text has been added to the results to clarify this. The non-uniform distribution of Klf7 across cortical layers at P2 and P7 likely indicates that that KLF switch, while conserved, occurs with variable kinetics across cortical cell types which could reflect their distinct rates of maturation in vivo (Gao et al., 2025). Minor edits have been made to the text associated with Figure 2 to call attention to this detail.

(2) Figure 6C1 describes three clusters of DEGs. If I understand correctly, cluster 1 represents downregulated DEGs by KLF6/7 that are also developmentally downregulated between P10 and P20 and cluster 2 represents downregulated DEGs by KLF6/7 that do not change between P10 and P20. It could be interesting to see how these gene sets compare with one another. For example, are the genes in cluster 1 that decrease from P10 to P20 more related to axon regulation/cytoskeleton when compared to cluster 2 which consist of genes that do not change from P10 to P20 and perhaps may reflect other regulatory functions of KLF6/7.

We appreciate this suggestion and we have updated Figure 6C1 to reflect this observation by highlighting genes that are part of significant enriched GO terms to allow the reader to see which cluster they belong to. Separate GO analyses performed for each downregulated gene cluster did not yield significant results and were therefore not included.

**Reviewer #3 (Recommendations for the authors):**

See above

Additional points:

(1) Upstream transcriptional regulation of KLFs:

The idea that KLFs are regulated by Thyroid hormone (T3) is interesting, and the manuscript would be strengthened by exploring this idea further. This could be done, e.g. by analyzing existing snRNA-seq on genes regulated in cortical neurons by T3 (see PMID: 39178853). Similarly, KLFs were suggested to act together with AP1, and many KLFs were previously found to be activity-regulated - hence, the manuscript would be strengthened by analyzing the connection between KLFs and AP1. This could be done e.g. by re-analyzing transcriptomic and proteomic data generated e.g. by the Greenberg laboratory.

We agree with the reviewer that the role of T3 in driving the KLF switch is intriguing and is an active area of investigation in our lab. However, these experiments are ongoing and are beyond the scope of this current manuscript. A preliminary analysis of the T3-treated snRNA-seq dataset present in Hochbaum et al., 2024 found that all major excitatory cortical cell types upregulate Klf9 upon T3 treatment while the expression of selected shared targets including Dpysl3 and Gap43 are downregulated in several cell types, supporting our hypothesis. These findings have been included in Figure S9.

It was not our intention to suggest that there may be cooperation between AP1 and the KLF family, as AP1 motif enrichment was detected in shared upregulated genes while the KLF/Sp motif was found in shared downregulated genes. Furthermore, DEGs from both KLF knockdown experiments had exclusive enrichment for promoter KLF/Sp motifs, making it unlikely that there is widespread co-regulation of these genes by the AP1 family.

(2) Figure 5D and Page 27, regarding Rac3:

The authors state that " no change in accessibility in motif-positive peaks upstream of Plppr1 and Rac3 (Figures 5D and S5C)." This statement seems incorrect for Rac3 in L6 neurons where the ATAC signal is strongly reduced at P30 relative to P2. The authors might want to explain this or choose a better example.

We acknowledge that Rac3 was a poor choice to represent a developmentally regulated Klf9/13 target with no change in promoter accessibility, and have updated Figure 5 with Atat1, a tubulin acetylase and shared KLF target, as our exemplar for this category of transcript.

(3) Supplemental Figure 2:

There seems to be a discrepancy between the expression levels in Panels A and C: the data in Panel A were generated from adult mouse cortex, and the levels of six KLFs are indicated as rather high - however, in Panel C, the adult levels of KLF6, 7 and 13 are rather low. How can this be explained?

We thank the reviewer for detecting this apparent discrepancy. Most of this can be attributed to the log scale used in Figure S2A, which flattens expression differences in the moderate to high expression range. We elected to use a log scale in Figure S2A to highlight KLF family members with higher cortical expression relative to those with low or no expression. In addition, the counts displayed in this figure were batch-corrected to account for significant batch effects between libraries from additional excitatory cortical cell types included in Figure S2A, which were prepared and sequenced at different locations (Brandeis University v. Janelia Research Campus, as in Sugino et al., 2019). The additional processing step has been included in our methods section.

(4) IGV plots in Figure 5D and Supplemental Figure 5B:

It might be worth highlighting/shading the promoter region to see the position of the peaks relative to the promoters and TSSs.

The TSS is indicated on all IGV plots by a dark arrow indicating the direction of transcription, so the promoter can be inferred to be immediately upstream.

(5) Middle of page 20:

Remove "saw"; this seems like a typo.

(6) Page 27, reference to the plot for Plppr1:

The plot for Plppr1 is in Supplemental Figure 5B2, not in S5C.

(7) Page 28:

There seems to be a typo/omission before "...general applicability of CRISPRi"
